# Quantitative proteomics reveals protein dysregulation during T cell activation in multiple sclerosis patients compared to healthy controls

**DOI:** 10.1186/s12014-022-09361-1

**Published:** 2022-07-05

**Authors:** Chiara Cappelletti, Anna Eriksson, Ina Skaara Brorson, Ingvild S. Leikfoss, Oda Kråbøl, Einar August Høgestøl, Valeria Vitelli, Olav Mjaavatten, Hanne F. Harbo, Frode Berven, Steffan D. Bos, Tone Berge

**Affiliations:** 1 Department of Mechanical, Electronics and Chemical Engineering, Faculty of Technology, Art and Design, OsloMet—Oslo Metropolitan University, Postboks 4, St. Olavs Plass, 0130 Oslo, Norway; 2 Neuroscience Research Unit, Department of Research, Innovation and Education, Oslo University Hospital, Oslo, Norway; 3 Institute of Clinical Medicine, Medical Faculty, University of Oslo, Oslo, Norway; 4 Department of Neurology, Oslo University Hospital, Ullevål, Postboks 4950, 0424 Nydalen, Oslo, Norway; 5 Department of Psychology, Faculty of Social Sciences, University of Oslo, Oslo, Norway; 6 Oslo Centre for Biostatistics and Epidemiology, Department of Biostatistics, University of Oslo, Oslo, Norway; 7 Proteomics Unit at University of Bergen (PROBE), Department of Biomedicine, University of Bergen, Postboks 7804, 5020, Bergen, Norway

**Keywords:** Autoimmunity, Multiple sclerosis, T cell activation, Proteomics, Disease susceptibility genes

## Abstract

**Background:**

Multiple sclerosis (MS) is an autoimmune, neurodegenerative disorder with a strong genetic component that acts in a complex interaction with environmental factors for disease development. CD4^+^ T cells are pivotal players in MS pathogenesis, where peripherally activated T cells migrate to the central nervous system leading to demyelination and axonal degeneration. Through a proteomic approach, we aim at identifying dysregulated pathways in activated T cells from MS patients as compared to healthy controls.

**Methods:**

CD4^+^ T cells were purified from peripheral blood from MS patients and healthy controls by magnetic separation. Cells were left unstimulated or stimulated in vitro through the TCR and costimulatory CD28 receptor for 24 h prior to sampling. Electrospray liquid chromatography-tandem mass spectrometry was used to measure protein abundances.

**Results:**

Upon T cell activation the abundance of 1801 proteins was changed. Among these proteins, we observed an enrichment of proteins expressed by MS-susceptibility genes. When comparing protein abundances in T cell samples from healthy controls and MS patients, 18 and 33 proteins were differentially expressed in unstimulated and stimulated CD4^+^ T cells, respectively. Moreover, 353 and 304 proteins were identified as proteins exclusively induced upon T cell activation in healthy controls and MS patients, respectively and dysregulation of the Nur77 pathway was observed only in samples from MS patients.

**Conclusions:**

Our study highlights the importance of CD4^+^ T cell activation for MS, as proteins that change in abundance upon T cell activation are enriched for proteins encoded by MS susceptibility genes. The results provide evidence for proteomic disturbances in T cell activation in MS, and pinpoint to dysregulation of the Nur77 pathway, a biological pathway known to limit aberrant effector T cell responses.

## Background

Multiple sclerosis (MS) is a complex autoimmune disorder with a significant health and societal burden [1, 2]. It is a chronic inflammatory, demyelinating disorder of the central nervous system (CNS) that leads to both cognitive and physical deficits [1, 3]. Introduction of disease modifying treatments has ameliorated the conditions of many patients [4], but development of personalized health care is partly precluded due to poor understanding of the biological processes underlying the disease. In addition to major genetic risk variants located in the HLA-gene region, genome-wide association studies (GWAS) have identified additional 200 autosomal MS-associated single nucleotide polymorphisms (SNPs). These findings combined with gene expression profiles have highlighted the importance of several peripheral immune cell types for MS onset, including both the innate and the adaptive immune response [5–7]. CD4^+^ T cells are important regulators of the adaptive immune system and have long been considered to play pivotal roles in MS pathogenesis [8], in which peripheral activation results in migration of these cells into the CNS, leading to demyelination and axonal degeneration [9].

Genome-wide studies on epigenetic modifications (e.g. DNA methylation) and gene expression of whole blood, peripheral blood mononuclear cells (PBMCs) and immune cell subtypes have been conducted to investigate potential immune dysregulation in MS. With few exceptions, no overlap was observed between the studies [10–23]. Moreover, it is becoming increasingly clear that the correlation between mRNA and protein copy numbers varies widely [24, 25], and proteomic studies are therefore needed to complement and confirm findings at the epigenetic or gene expression level. Quantitative high-resolution mass spectrometry-based proteomics enables system-wide studies at the protein level; however, such studies are scarce in samples from individuals with complex diseases such as MS.

We have recently performed this approach on CD4^+^ and CD8^+^ T cells freshly purified from blood in a small cohort of MS patients and healthy controls (HCs) [26]. In our proteomic data set, we found an enrichment of proteins involved in T-cell specific activation in CD4^+^ T cells among the proteins differentially expressed between MS patients and HCs, which was not observed in CD8^+^ T cells [26], prompting us to investigate T-cell activation in CD4^+^ T cells. Importantly, our proteomic study, as well as other studies at the epigenetic and gene expression level, were performed on unstimulated cells and represents an image of the state of the cells at the time of harvesting. Novel disease-associated pathways could be identified if cells were activated prior to proteomic profiling, as illustrated at the RNA level for MS and coeliac disease, by Hellberg et al. [27] and Quinn et al. [28], respectively.

Using liquid chromatography combined with tandem mass spectrometry, we performed quantitative proteomics of CD4^+^ T cells from relapsing–remitting MS (RRMS) patients and HCs. Cells were left unstimulated or stimulated through the T cell receptor (TCR) in vitro allowing us to disentangle potential CD4^+^ T cell specific differences induced by T cell activation, providing novel insights into disease mechanisms of MS.

## Materials and methods

### MS patients and healthy controls

Blood samples were collected from 20 untreated female RRMS patients (mean age 36.7 years, range 21–63 years) with median extended disability status scale (EDSS) score of 1.5 (range 0–5.5) and mean disease duration of 8 years (range 0.5–38). For one of the patients, the EDSS score was assessed by inspection of their medical journals. HC samples were collected from 20 age- and sex-matched individuals (mean age 37.0 years, range 23–50 years). See Table 1 for summary statistics and demographic information on the MS cohort. All participants were of self-declared Nordic ancestry, and the HCs reported no MS in close family members. MS patients were recruited from the MS out-patient clinic at Oslo University Hospital, Norway, and the HCs from the patients’ social networks and among hospital employees. All MS patients fulfilled the updated McDonald criteria for MS at their time of diagnosis [29]. At the time of sample collection, the included individuals did not have any ongoing infection, and the MS patients had not experienced a relapse, or received steroids for at least three months prior to enrollment. The Regional Committee for Medical and Health Research Ethics South East, Norway approved the study. Written informed consent was obtained from all study participants.

**Table 1:** Characteristics of individual MS patients and summaries of patients and healthy controls

Patient	Age	Disease duration	EDSS
MS1	44	13	0.0
MS2	45	18	2.0
MS3	63	38	5.5
MS4	30	8	3.5
MS5	39	9	1.5
MS6	31	6	1.5
MS7	32	6	2.0
MS8	41	3	0.0
MS9	29	1.5	4.0
MS10	21	0.5	1.5
MS11	37	2	1.5
MS12	39	5	2.5
MS13	37	12	1.5
MS14	44	2	1.0
MS15	37	6	2.5
MS16	25	0.8	1.5
MS17	29	15	3.5
MS18	30	0.5	1.0
MS19	52	19	1.5
MS20	28	1	1.5
Summarized			
Patients mean or median * (range)	36.65 (21–63)	8.31 (0.5–38)	2.0 * (0.0–5.5)
Healthy controls mean (range)	36.95 23–50	N/A	N/A
p-value	0.92		

The table includes demographic data for each individual MS patient at inclusion, with age and disease duration in years

*EDSS* expanded disability status scale, *N/A* not applicable

### Isolation of human CD4^+^ T cells

Peripheral blood mononuclear cells were isolated from whole blood using density gradient centrifugation with Lymphoprep™ (Axis Shield, Dundee, Scotland), before negative selection of CD4^+^ T cells with EasySep™ Human CD4^+^ T Cell Isolation Kit (STEMCELL Technologies, Vancouver, Canada). Cell purity was measured by flow cytometry (Attune Acoustic Focusing Flow Cytometer, Life Technologies, Carlsbad, CA, USA or FACSCalibur, BD Biosciences, Franklin Lakes, NJ, USA) using the fluorescein isothiocyanate-conjugated (FITC) mouse anti-human CD4 antibody (clone RTF-4 g) and mouse IgG1 isotype control (15H6) (both from Southern Biotech, Birmingham, AL, USA). Aliquots of CD4^+^ T cells were subsequentially frozen with dimethyl sulfoxide (DMSO) (Sigma-Aldrich®, Darmstadt, Germany) and stored in liquid nitrogen until usage.

### T cell activation

Live CD4^+^ T cells stored in liquid nitrogen were thawed and left unstimulated in X-VIVO 15 medium (Lonza, Basel, Switzerland) or stimulated in 96-well plates coated with 5 µg/ml anti-CD3 (mouse anti-human CD3, Clone OKT3, eBioscience™ by Thermo Fisher Scientific, San Diego, CA, USA) in X-VIVO 15 medium supplemented with 2 µg/ml anti-CD28 (purified NA/LE mouse anti-human CD28, BD Biosciences). Cells were cultured at a starting density of 1 million cells/ml for 24 h at 37 °C and 5% CO_2_. Cell pellets of 200,000 cells from each sample were kept at − 80 °C until preparation for mass spectrometry analyses. An aliquot of unstimulated and stimulated CD4^+^ T cells were stained with FITC-conjugated mouse anti-human CD69 antibody or mouse IgG1 isotype control (both from ImmunoTools, Friesoythe, Germany) prior to staining with the LIVE/DEAD™ Fixable Far Red Dead Stain Kit (Invitrogen, by Thermo Fisher Scientific, Carlsbad, CA, USA) for flow cytometry analysis using FACS Canto II flow cytometer (BD Biosciences) to evaluate cell activation and viability. Analysis of flow cytometry data was performed with FCS Express 6 Flow Cytometry Software 2.1 (De Novo Software, Glendale, CA, USA).

### Sample preparation and protein digestion

The frozen cell pellets were solubilized in 40 μl ice-cold RIPA buffer, containing 1% NP40, 50 mM TrisHCl pH 7.6, 0.5% sodium deoxycholate, 0.1% SDS, 150 mM NaCl, and 1 × cOmplete™ EDTA-free protease inhibitor (cat. No. 11873580001, Roche). Samples were homogenized on ice for 15 min followed by four cycles of ultra-sonification in ice-cold water with 30 s on and 30 s off, followed by another 15 min on ice. After centrifugation for 10 min at 16,200×*g* at 4 °C, supernatants were collected. Protein concentrations in the lysates were measured by Pierce BCA protein assay (Thermo Fisher Scientific, Rockford, IL, USA) and the absorbance values at 562 nm were obtained by Multiscan FC 3.1 ELISA reader (Thermo Fisher Scientific, Rockford, IL, USA). Subsequently, 2 μl 150 mM dithiothreitol (DTT) were added to 6 µg protein in 25 µl RIPA buffer for cysteine reduction and incubated for 1 h at RT. Cysteines were alkylated after addition of 3 µl 300 mM iodoacetamide (1 h, at room temperature protected from light). Digestion of proteins was accomplished using the SP3 protocol [30] with a few modifications: 2 µl (65 µg) magnetic beads (Sera-Mag SpeedBeads, GE Healthcare, cat. no. 45152105050250 and 65,152,105,050,250) were added to the sample, and the protein binding/aggregation with the beads was accomplished by adding ethanol to 70% final concentration. After thorough washing in 80% ethanol, the protein/beads pellet was digested with trypsin (sequencing grade-modified trypsin from Promega, GmbH, Mannheim, Germany) dissolved in 50 µl 100 mM ammonium bicarbonate with a trypsin-to-protein ratio of 1:25. Samples were incubated at 37 °C for 16 h at 1000 rpm. Tryptic peptides were collected, and beads washed once with 50 µl 0.5 M NaCl. Sample cleanup was performed using a reverse-phase OasisR HLB μElution Plate 30 μm (2 mg HLB sorbent, Waters, Milford, MA). After lyophilization, the dried peptides were suspended in 12 μl of 0.5% formic acid containing 2% acetonitrile. Two μl were used for protein quantification based on absorbance at 280 nm using a NanoDrop spectrophotometer (Thermo Fisher Scientific, Carlsbad, CA, USA), and 0.6 μg of the mixture were analyzed with mass spectrometry.

### Liquid chromatography-mass spectrometry/mass spectrometry analysis

Peptides were analyzed by electrospray liquid chromatography–tandem mass spectrometry (LC–MS/MS) using a quadrupole–orbitrap instrument (QExactive HF, Thermo Fisher Scientific, Carlsbad, CA, USA). The LC run length of 3 h was performed on a 50 cm analytical column (PepMap RSLC, 50 cm × 75 µm ID EASY-spray column, packed with 2 µm C18 beads (Thermo Fisher Scientific, Carlsbad, CA, USA)). Peptides were loaded and desalted on a pre-column (Acclaim PepMap 100, 2 cm × 75 µm ID nanoViper column, packed with 3 µm C18 beads (Thermo Fisher Scientific, Carlsbad, CA, USA)) with 0.1% (v/v) trifluoroacetic acid, and eluted with a gradient composition as follows: 5% B during trapping (5 min) followed by 5–8% B for 0.5 min, 8–24% B for the next 109.5 min, 24–35% B over 25 min, and 35–80% B over 15 min. Elution of very hydrophobic peptides and conditioning of the column were performed during 15 min isocratic elution with 80% B and 20 min isocratic elution with 5% B respectively. Mobile phases A and B contained 0.1% formic acid (vol/vol) in water and 100% acetonitrile, respectively, and the flow rate was 200 nl per min. A full scan in the mass area (m/z) of 375–1500 was performed in the Orbitrap. For each full scan performed at a resolution of 120,000 (m/z 200), the 12 most intense ions above an intensity threshold of 50,000 counts, and charge states 2 to 5 were sequentially isolated and fragmented in the Higher-Energy Collision Dissociation (HCD) cell. Fragmentation was performed with a normalized collision energy (NCE) of 28%, and fragments were detected in the Orbitrap at a resolution of 30,000 (m/z 200), with first mass fixed at m/z 100. One MS/MS spectrum of a precursor mass was allowed before dynamic exclusion for 25 s with “exclude isotopes” on. Lock-mass internal calibration (m/z 445.12003) was used.

### Mass spectrometry data analysis

Mass spectrometry (mass spec) raw files were analyzed by the Proteome Discoverer™ software (Thermo Fisher Scientific, Carlsbad, CA, USA, version 2.4), and peak lists were searched against the human SwissProt FASTA database (version May 2020), and a common contaminants database by Sequest HT and MS Amanda 2.0 search engines. Methionine oxidation and acetylation on protein N-terminus were added as variable modifications, while cysteine carbamidomethylation was used as fixed modification. False discovery rate (Percolator, http://percolator.ms/) was set to 0.01 for proteins and peptides (minimum length of six amino acids) and was determined by searching the reversed database. Trypsin was set as digestion protease, and a maximum of two missed cleavages were allowed in the database search. Mass recalibration was performed prior to peptide identification using precursor and fragment mass deviation of 20 ppm and 0.5 Da respectively. The main search was then conducted with an allowed mass spec and mass spec/mass spec mass deviation tolerance of 10 ppm and 0.02 Da respectively. Retention time alignment and detection of precursor features across samples were done using the Minora Feature Detector node in Proteome Discoverer™.

### Data processing

A total of 6687 proteins were identified by the Proteome Discoverer™ 2.4 Software (Thermo Fisher Scientific, Carlsbad, CA, USA). Of these, 178 protein signals were marked as contaminants and therefore removed from further analysis. In Perseus (Perseus Software, version 1.5.6.0), the normalized abundances from Proteome Discoverer™ were log2 transformed and the normal distributions were controlled by plotting the histograms. Proteins with valid values in at least 70% of the samples in at least one of the four groups (HC: unstimulated, HC: stimulated, MS: unstimulated and MS: stimulated) were used for analysis. The missing protein abundances were imputed from the normal distribution using default settings in Perseus.

### Statistical analyses

All analyses presented were performed using the R software version 4.0.4. Differences in protein abundances upon T cell activation were assessed using a paired two-tailed Student’s *t*-test. When comparing protein abundance between samples from MS patients and HCs, a Welch´s test (for unequal variances) was used. Principal component analysis (PCA) plots were generated using protein intensities of differentially expressed proteins as variables. For each PCA, the cutoff to define the most influential loadings in determining the corresponding score value was calculated as the square root of one divided by the number of variables; this cutoff value corresponds to the assumption of uniform contribution of all loadings. For validation analysis, 100 discovery cohorts were simulated by randomly selecting ten MS samples and ten HC samples and the differentially expressed proteins identified in these simulated cohorts were used as input for performing PCA in the remaining samples.

Within each analysis stratum, the Benjamini-Hochberg (B-H) procedure was used to correct for multiple testing and adjusted p-values considered significant are indicated in the results section.

### Ingenuity pathway analysis

QIAGEN´s Ingenuity^®^ pathway Analysis (IPA^®^ QIAGEN, version 52,912,811, date: 2020-09-07) was used for functional interpretation of significantly expressed proteins. The default settings were used, species was set to “all” and “T lymphocytes”, “Immune cell lines”, “CCRF-CEM”, “Jurkat” and “MOLT-4” were selected among the tissues and cell lines. A Benjamini-Hochberg (B-H) multiple testing correction was used, and a value below 0.05 (-log (B-H p-value) > 1.3) was considered significant.

## Results

### Protein dysregulation is observed in CD4^+^ T cells from MS patients

**Fig. 1:**
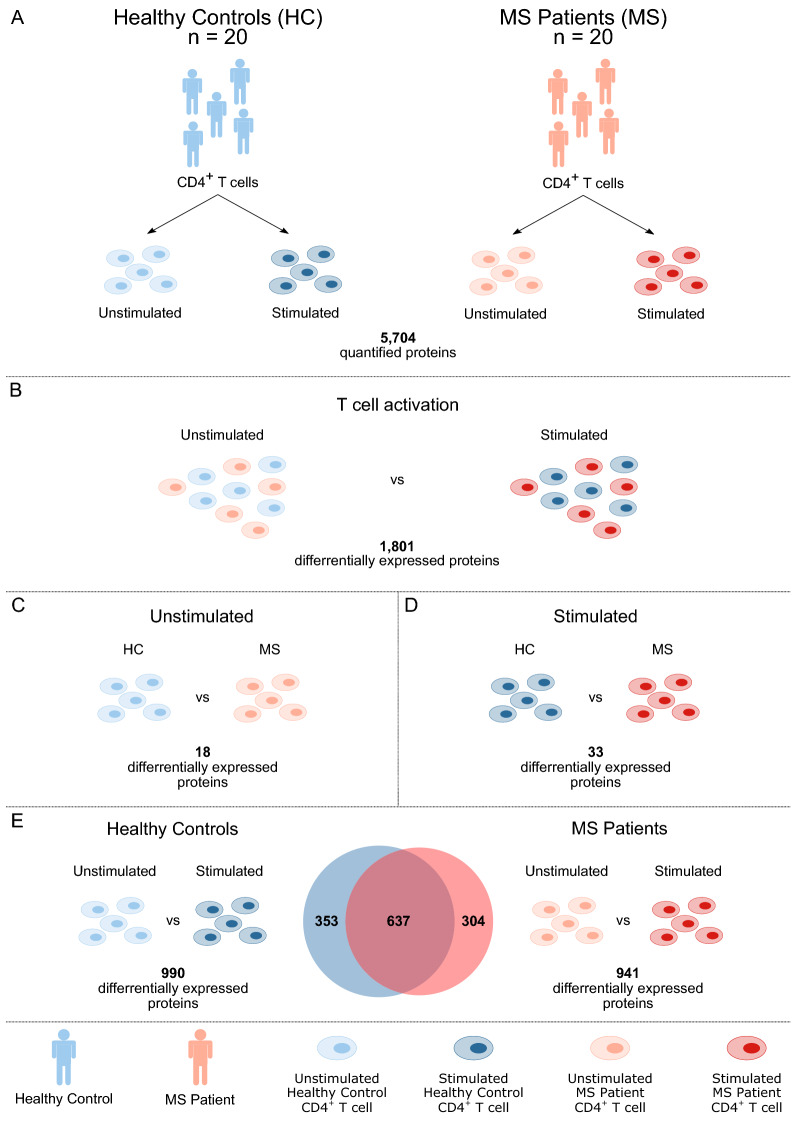
An overview of the study. Study design (**A**). Differentially expressed proteins between unstimulated and stimulated CD4^+^ T cells (**B**). Differentially expressed proteins between HC and MS in unstimulated CD4^+^ T cells (**C**) and in stimulated CD4^+^ T cells (**D**). Proteins that change in abundance upon CD4^+^ T cell activation of samples from MS and HC (**E**). The Venn diagram displays the number of proteins that were differentially expressed between unstimulated and stimulated CD4^+^ T cells from HCs (blue) and MS patients (red)

**Fig. 2:**
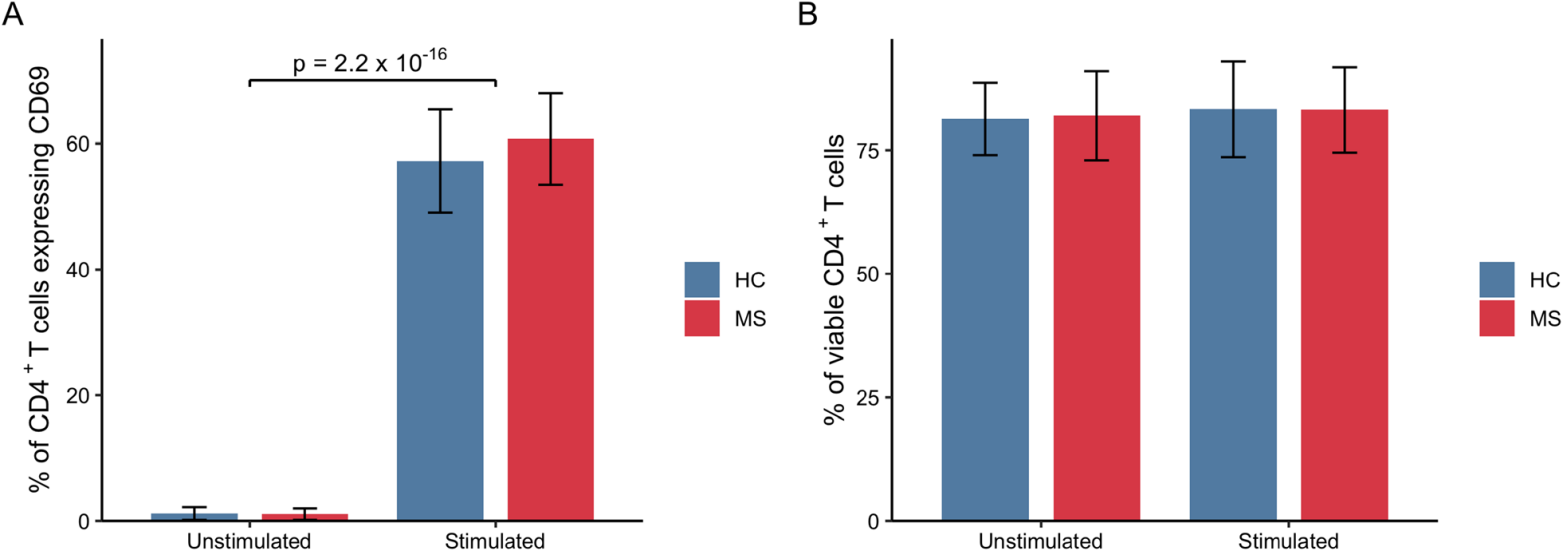
Flow cytometry characterization of CD4^+^ T cells from MS patients (MS) and healthy controls (HC). Proportions of (**A**) CD69^+^ and (**B**) viable CD4^+^ T cells in unstimulated and stimulated (anti-CD3/CD28 antibody stimulation) samples. Mean and standard deviation are shown, Mann Whitney U test showed no significant differences across groups (MS vs HC). *MS*  multiple sclerosis, *HC*  healthy controls

In this study, we examined the differences at the proteomic level of CD4^+^ T cells from RRMS patients (n = 20) and HCs (n = 20). CD4^+^ T cells were left unstimulated or stimulated through the TCR (anti-CD3; OKT3) and costimulatory CD28 receptor (anti-CD28) for 24 h prior to sampling (Fig. 1A). T cell activation was verified by measuring the cell surface expression of the T cell activation marker CD69 by flow cytometry (Fig. 2A). There were no significant difference in T cell activation nor cell viability between samples from MS patients and healthy controls (Fig. 2). Using a label-free proteomics approach, we were able to identify and quantify a total of 5704 proteins. Of these proteins, the abundance of 1,801 was changed upon T cell activation (adjusted p ≤ 0.01) (Fig. 1B).

**Fig. 3:**
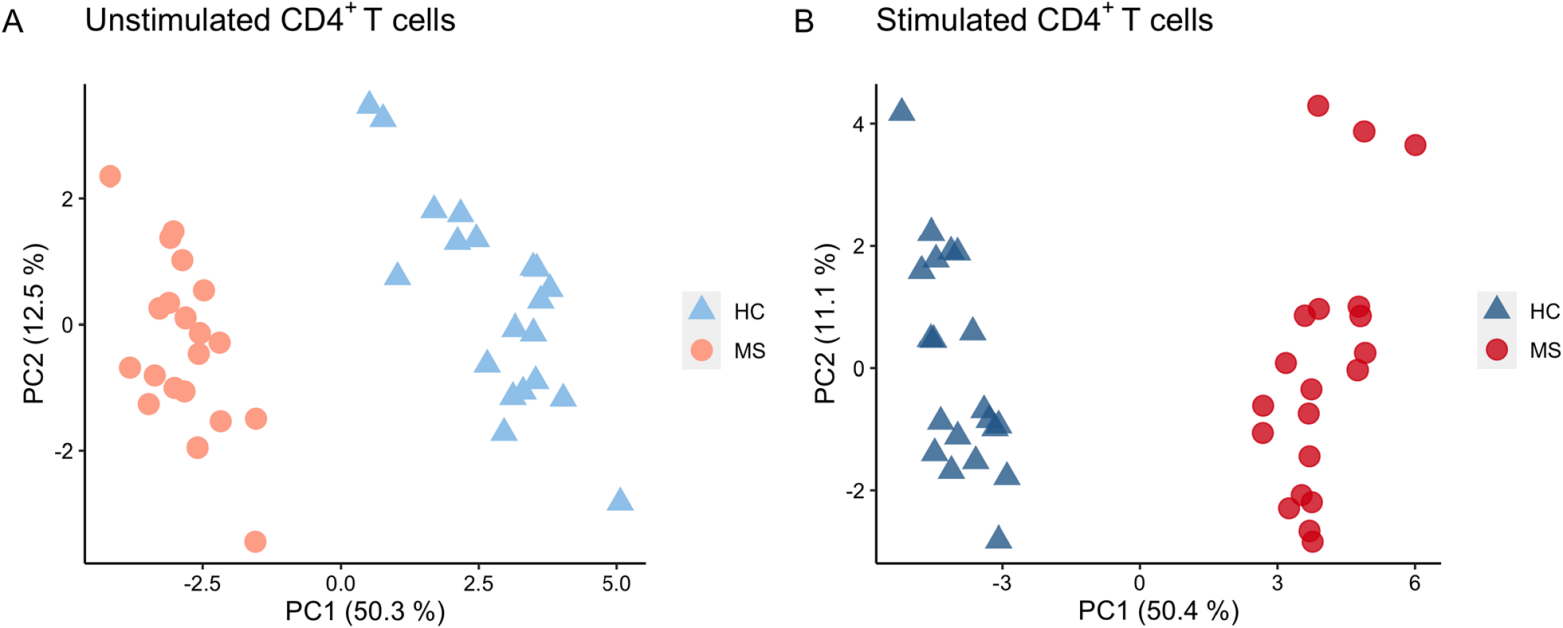
Principal component analysis (PCA) of differentially expressed proteins. Scores of the first (PC1) and second (PC2) principal components obtained by PCA of proteins significantly different in abundance (p ≤ 0.05) between MS patients (MS; red circles) and HCs (HC; blue triangles) in (**A**) unstimulated and (**B**) stimulated CD4^+^ T cells

When comparing protein abundances in the T cell samples from HCs and MS patients, 18 and 33 proteins were differentially expressed (adjusted p ≤ 0.05) in unstimulated (Table 2, Fig. 1C) and stimulated CD4^+^ T cells (Table 3, Fig. 1D), respectively, with two proteins; diphthamide synthetase, encoded by *DPH6*, and enhancer of polycomb homolog 1, encoded by *EPC1,* being significant in both conditions. Diphthamide synthetase expression was higher in unstimulated cells from MS patients (log2 fold change = 3.30), whereas its expression was lower in stimulated cells from MS patients (log2 fold change = − 1.91), compared to HC. Enhancer of polycomb homolog 1 showed higher fold change between MS and HC samples in stimulated samples (log2 fold change = 3.47) compared to unstimulated samples (log2 fold change = 2.34). The principal component analysis (PCA) plots of significant proteins in each analysis show separated clusters of samples from MS patients and HCs in unstimulated (Fig. 3A) and stimulated (Fig. 3B) CD4^+^ T cells. Moreover, the PCA plot of the stimulated CD4^+^ T cells shows two clusters for the MS samples, one main cluster composed of 17 samples and the second cluster of three. Close to 50% of the total variation in the dataset, which captures the separation between MS and HCs, was explained by the first component, whereas the second component, which captures the separation between the two MS clusters for the stimulated samples, explained 11–12% of the variance in each analysis. The loadings of the first two principal components for each PCA are shown in Tables 2 and 3, for unstimulated and stimulated CD4^+^ T cells, respectively. The loadings that contribute the most to the score values of PC2 shown in Fig. 3B for the stimulated samples were detected by comparison to a cutoff value (cutoff = 0.174), and these influential loadings correspond to 15 of the 33 differentially expressed proteins between MS and HCs in the stimulated samples (highlighted in bold in Table 2). These 15 proteins thus have a strong effect on PC2, and greatly influence the separation in the samples creating the two MS clusters in Fig. 3B.

**Table 2:** Differentially expressed proteins in unstimulated CD4^+^ T cells

Accession	Protein identity	Gene names	p-value	Adjusted p-value	FC MS versus HC (log2)	Median intensity MS (log2)	MS SD	Median intensity HC (log2)	HC SD	% seq cov	# pep	PC1	PC2
Q7L8W6	Diphthine–ammonia ligase	*DPH6*	2.61E−13	1.49E−09	3.301	24.018	1.032	21.159	0.819	7	2	**− 0.253**	0.067
Q8TDQ7	Glucosamine-6-phosphate isomerase 2	*GNPDA2*	2.21E−09	6.31E−06	− 1.851	25.017	0.660	26.798	0.817	64	12	0.156	**0.384**
Q9UKU7	Isobutyryl-CoA dehydrogenase, mitochondrial	*ACAD8*	6.47E−09	1.23E−05	1.028	26.741	0.283	25.805	0.501	37	11	0.174	**0.331**
Q92828	Coronin-2A	*CORO2A*	4.86E−08	6.93E−05	2.265	23.155	1.130	20.378	0.964	6	3	0.192	**0.366**
A8MW92	PHD finger protein 20-like protein	*PHF20L1*	4.32E−07	4.86E−04	1.364	23.108	0.596	21.700	0.787	2	3	**− 0.261**	0.146
Q9HD15	Steroid receptor RNA activator 1	*SRA1*	5.11E−07	4.86E−04	1.491	23.969	0.589	22.233	0.895	23	5	**− 0.255**	0.133
Q9H2F5	Enhancer of polycomb homolog 1	*EPC1*	9.04E−07	7.36E−04	2.343	23.484	0.405	20.419	1.496	1	1	**− 0.292**	0.081
Q12923	Tyrosine-protein phosphatase nonreceptor type 13	*PTPN13*	1.37E−06	9.75E−04	− 1.266	25.186	0.683	26.405	0.715	0	1	**− 0.245**	**0.297**
Q8TCE6	DENN domain-containing protein 10	*DENND10*	2.83E−06	1.79E−03	2.039	25.989	1.239	23.524	1.099	24	6	**− 0.263**	0.108
O43504	Ragulator complex protein LAMTOR5	*LAMTOR5*	1.64E−05	8.96E−03	− 1.583	21.906	0.915	23.469	1.097	37	2	− 0.228	**0.256**
Q9H7Z3	Nuclear exosome regulator NRDE2	*NRDE2*	1.73E−05	8.96E−03	− 2.441	19.733	1.071	22.690	1.857	2	2	**− 0.256**	− 0.045
Q16401	26S proteasome non-ATPase regulatory subunit 5	*PSMD5*	2.14E−05	9.39E−03	0.577	29.015	0.397	28.500	0.353	73	27	**− 0.243**	0.135
Q9HD26	Golgi-associated PDZ and coiled-coil motifcontaining protein	*GOPC*	2.03E−05	9.39E−03	− 1.638	22.742	1.172	24.340	0.927	18	7	0.162	**0.385**
Q12797	Aspartyl/asparaginyl beta-hydroxylase	*ASPH*	2.91E−05	1.19E−02	2.423	26.114	1.155	22.808	1.895	4	3	**− 0.238**	**0.254**
O75764	Transcription elongation factor A protein 3	*TCEA3*	7.49E−05	2.85E−02	− 1.026	22.367	0.761	23.294	0.698	21	6	0.226	**0.302**
P08134	Rho-related GTP-binding protein RhoC	*RHOC*	1.04E−04	3.70E−02	− 2.197	20.231	1.385	23.063	1.776	69	12	**0.249**	-0.100
P29597	Non-receptor tyrosine-protein kinase TYK2	*TYK2*	1.19E−04	3.78E−02	1.144	21.722	0.858	20.650	0.829	1	2	0.194	**0.238**
Q96GS4	BLOC-1-related complex subunit 6	*BORCS6*	1.18E−04	3.78E−02	0.685	23.655	0.465	23.067	0.539	42	8	**− 0.294**	-0.046

The table shows the accession number, protein identity and gene names for each protein, in addition to the unadjusted (p-value) and adjusted p-value, the log2-fold changes in MS versus HC based on normalized values, median log2-transformed protein abundances with standard deviation (SD) for each group, the percentage of sequence coverage (% seq cov), the number of peptides (# pep) identified for each protein, and the loadings for the first (PC1) and the second principal component (PC2). Large loadings (cutoff 0.236) are highlighted in bold

**Table 3:** Differentially expressed proteins in stimulated CD4^+^ T cells

Accession	Protein identity	Gene names	p-value	Adjusted p-value	FC MS vsrses HC (log2)	Median intensity MS (log2)	MS SD	Median intensity HC (log2)	HC SD	% seq cov	# pep	PC1	PC2
Q13588	GRB2-related adapter protein	*GRAP*	1.77E−16	1.01E−12	5.346	31.399	1.303	25.885	0.832	61	10	**0.224**	− 0.070
Q9H2F5	Enhancer of polycomb homolog 1	*EPC1*	5.59E−15	1.59E−11	3.473	23.533	0.548	20.114	0.946	1	1	**0.213**	− 0.095
Q6ICL3	Transport and Golgi organization protein 2 homolog	*TANGO2*	2.05E−11	3.81E−08	4.479	26.502	0.813	22.015	1.666	23	4	0.156	**− 0.257**
Q9BV20	Methylthioribose-1-phosphate isomerase	*MRI1*	2.67E−11	3.81E−08	− 2.355	25.694	0.521	27.987	0.897	36	11	**0.198**	− 0.122
Q6UW02	Cytochrome P450 20A1	*CYP20A1*	6.87E−11	7.83E−08	− 1.365	26.049	0.540	27.365	0.355	55	21	0.143	**− 0.210**
Q8WV92	MIT domain-containing protein 1	*MITD1*	8.61E−10	8.18E−07	− 3.449	21.702	1.336	25.776	1.359	15	3	0.145	**− 0.313**
Q9H4A6	Golgi phosphoprotein 3	*GOLPH3*	1.75E−09	1.43E−06	2.248	26.038	1.023	23.927	0.456	26	7	**0.229**	− 0.028
Q9NWS0	PIH1 domain-containing protein 1	*PIH1D1*	3.57E−09	2.55E−06	− 1.367	24.477	0.552	25.772	0.581	38	7	0.148	**− 0.299**
Q9Y5B6	PAX3- and PAX7-binding protein 1	*PAXBP1*	9.10E−08	5.77E−05	1.162	26.019	0.335	24.924	0.650	22	16	− 0.137	− 0.013
Q96G46	tRNA-dihydrouridine(47) synthase [NAD(P)( +)]-like	*DUS3L*	1.14E−07	5.93E−05	− 2.660	22.773	1.476	25.446	0.966	23	10	**0.186**	**− 0.202**
Q9BRQ6	MICOS complex subunit MIC25	*CHCHD6*	1.04E−07	5.93E−05	− 4.215	21.766	0.898	26.389	2.373	20	4	**0.174**	− 0.091
Q7L8W6	Diphthine–ammonia ligase	*DPH6*	2.76E−07	1.31E−04	− 1.913	21.418	0.980	23.404	0.962	7	2	**− 0.183**	− 0.173
Q8WUK0	Phosphatidylglycerophosphatase and protein-tyrosine phosphatase 1	*PTPMT1*	3.13E−07	1.37E−04	− 3.168	20.553	1.865	24.854	1.171	6	1	− 0.142	**− 0.184**
Q02539	Histone H1.1	*H1-1*	4.31E−07	1.76E−04	− 3.260	22.786	1.968	26.447	1.160	50	15	0.164	− 0.114
Q2TBE0	CWF19-like protein 2	*CWF19L2*	2.63E−06	9.98E−04	− 1.787	21.730	1.185	23.318	0.757	6	5	− 0.164	**− 0.218**
Q5T4S7	E3 ubiquitin-protein ligase UBR4	*UBR4*	3.57E−06	1.27E−03	0.815	30.188	0.564	29.497	0.298	29	117	− 0.137	**− 0.224**
P54098	DNA polymerase subunit gamma-1	*POLG*	4.04E−06	1.28E−03	− 1.351	20.008	0.942	21.367	0.504	2	3	0.162	**− 0.307**
Q9H2K8	Serine/threonine-protein kinase TAO3	*TAOK3*	3.93E−06	1.28E−03	0.710	27.728	0.461	27.239	0.358	36	31	**− 0.174**	**− 0.192**
P17706	Tyrosine-protein phosphatase nonreceptor type 2	*PTPN2*	5.97E−06	1.79E−03	0.847	24.107	0.409	23.441	0.578	23	8	**− 0.204**	0.026
Q5THJ4	Vacuolar protein sorting-associated protein 13D	*VPS13D*	1.58E−05	4.49E−03	3.492	24.517	2.648	19.778	1.488	1	2	− 0.147	− 0.155
Q8IV53	DENN domain-containing protein 1C	*DENND1C*	1.88E−05	5.10E−03	− 0.596	25.891	0.445	26.401	0.295	35	20	− 0.143	− 0.044
O94762	ATP-dependent DNA helicase Q5	*RECQL5*	3.96E−05	1.03E−02	1.558	21.522	0.917	19.793	1.172	1	1	− 0.157	− 0.132
Q2M296	Methenyltetrahydrofolate synthase domain-containing protein	*MTHFSD*	4.59E−05	1.14E−02	− 3.585	26.603	3.050	31.143	0.892	11	3	**− 0.186**	**− 0.191**
Q96SB8	Structural maintenance of chromosomes protein 6	*SMC6*	8.32E−05	1.98E−02	− 1.074	23.137	0.690	24.224	0.838	11	9	− 0.144	**− 0.226**
Q8NEM7	Transcription factor SPT20 homolog	*SUPT20H*	1.00E−04	2.28E−02	− 1.168	20.132	0.830	21.584	0.869	2	2	− 0.168	0.051
Q8WXA9	Splicing regulatory glutamine/lysine-rich protein 1	*SREK1*	1.14E−04	2.50E−02	0.993	23.502	0.867	22.472	0.505	14	5	**− 0.202**	**− 0.174**
Q8IWV7	E3 ubiquitin-protein ligase UBR1	*UBR1*	1.41E−04	2.92E−02	0.511	26.361	0.313	25.872	0.433	21	31	**− 0.196**	− 0.009
Q9HC21	Mitochondrial thiamine pyrophosphate carrier	*SLC25A19*	1.44E−04	2.92E−02	− 1.415	20.523	1.025	22.021	1.090	11	4	0.152	**− 0.278**
Q5PSV4	Breast cancer metastasis-suppressor 1-like protein	*BRMS1L*	1.53E−04	3.01E−02	− 1.678	20.204	1.064	21.904	1.415	7	2	**− 0.177**	− 0.118
Q6WCQ1	Myosin phosphatase Rho-interacting protein	*MPRIP*	1.92E−04	3.66E−02	1.342	23.142	0.681	21.771	1.234	9	6	− 0.160	**− 0.184**
Q9BQ69	ADP-ribose glycohydrolase MACROD1	*MACROD1*	2.08E−04	3.82E−02	− 1.085	23.281	0.651	24.858	0.967	18	4	**− 0.182**	0.015
Q9H410﻿	Kinetochore-associated protein DSN1 homolog	*DSN1*	2.26E−04	4.03E−02	− 1.220	21.639	1.149	22.896	0.594	21	6	**− 0.176**	− 0.154
Q9BYC9	39S ribosomal protein L20, mitochondrial	*MRPL20*	2.43E−04	4.20E−02	− 0.643	23.390	0.533	24.157	0.468	24	4	**− 0.210**	− 0.070

The table shows the accession number, protein identity and gene names for each protein, in addition to the unadjusted (p-value) and adjusted p-value, the log2-fold changes in MS versus HC based on normalized values, median log2-transformed protein abundances with standard deviation (SD) for each group, the percentage of sequence coverage (% seq cov), the number of peptides (# pep) identified for each protein, and the loadings for the first (PC1) and the second principal component (PC2). Large loadings (cutoff 0.174) are highlighted in bold

### Validation of protein dysregulation in CD4^+^ T cells from MS patients by resampling

To validate the protein dysregulation observed in CD4^+^ T cells from MS patients, we simulated 100 discovery cohorts by randomly selecting ten MS samples and ten HC samples (n_MS_ = 10, n_HC_ = 10) for each simulated dataset. For both conditions (unstimulated and stimulated), we performed differential expression analysis in each of the 100 simulated discovery cohorts. We carried out PCA analysis based on the differentially expressed proteins (adjusted p ≤ 0.05) in each corresponding replication cohorts, consisting of the remaining samples (n_MS_ = 10, n_HC_ = 10). The number of significant proteins in the main analysis and the median number of significant proteins obtained from the validation analysis for each condition are listed in Table 4. The lower number of significant proteins found in the validation analysis is due to the lower power to detect differentially expressed proteins in smaller datasets (n = 10 versus n = 20). In the validation analysis, we found that the scores of the first principal components were statistically different (p ≤ 0.05) between MS and HC samples in 82% of the iterations for the unstimulated samples and in 61% of the iterations in the stimulated samples. Of note, in two out of 100 iterations in the unstimulated samples, no significant proteins were found, whereas in the validation analysis of the stimulated samples, significant proteins were found in all iterations. These analyses confirmed that most of the variance present in our samples captured by the first principal component was due to protein dysregulation in CD4^+^ T cells between MS patients and HCs.

**Table 4:** Numbers of significant differentially expressed proteins between MS patients (MS) and healthy controls (HC) in unstimulated and stimulated CD4^+^ T cells

	MS vs HC unstimulated	MS vs HC stimulated
Number of significant proteins in main analysis^a^	18	33
Median number of significant proteins in validation analysis with (range)^b^	2 (0–13)	10 (4–18)
^a^n = 20 in each group, ^b^n = 10 for each group per iteration, 100 iterations		

When comparing the differentially expressed proteins in samples from MS patients and HCs identified in the iteration analyses, we discovered that diphthamide synthetase, encoded by *DPH6*¸ was found in 98 iterations of the unstimulated samples, while Grb2-related adapter protein and enhancer of polycomb homolog, encoded by *GRAP* and *EPC1*, respectively, were found in all 100 iterations from stimulated samples.

### Proteins differentially expressed upon T cell activation are enriched for proteins expressed by MS-susceptibility genes

To test for enrichment of proteins encoded by MS susceptibility genes among the 1801 proteins whose abundance is changed upon T cell activation (Fig. 1B), the IDs of 285 most proximal genes were extracted from the list of 200 autosomal, non-HLA MS-associated SNPs [7]. For intergenic MS-associated SNPs, we extracted the most proximal genes both upstream and downstream of the SNPs. Out of these, 34 gene IDs corresponded to non-coding RNAs and were removed from the analysis. Not all MS susceptibility genes are expressed in T cells, and in our samples, we detected 97 proteins encoded by MS susceptibility genes that were expressed either in the unstimulated or stimulated samples. Of these, 43 proteins were among the 1,801 differentially expressed upon T cell activation regardless of the disease status. A Pearson’s Chi-squared test showed that there was a significant enrichment for proteins encoded by MS susceptibility genes among the 1801 proteins that were changed upon T cell activation (p = 0.0089; Table 5), highlighting the importance of this process in MS.

**Table 5:** Proteins differentially expressed upon T cell activation are enriched for proteins expressed by MS-susceptibility genes

	Proteins not expressed by MS susceptibility genes	Proteins expressed by MS susceptibility genes
Not differentially expressed upon T cell activation^a^	3849	54
Differentially expressed upon T cell activation^a^	1758	43
Pearson Chi-squared test p-value		0.0089

In the two-by-two table, the annotated and quantified proteins in our data set are divided into proteins encoded by MS susceptibility genes or not. Statistical testing of significance was performed according to Pearson’s Chi-squared test, and the p-value is given in the table

^a﻿^Proteins with Benjamini - Hochberg adjusted p - values ≤ 0.01 in the differential expression analysis between unstimulated and stimulated samples

### Ingenuity pathway analysis of differentially expressed proteins exclusively induced upon T cell activation in MS patients or in healthy controls

To elaborate on the differences in the T cell activation process in CD4^+^ T cells from MS patients and HCs, we specifically analyzed proteins that displayed a significant change in abundance upon T cell activation in HC and MS (Fig. 1E). We discovered 990 differentially expressed proteins (adjusted p ≤ 0.01) between unstimulated and stimulated CD4^+^ T cells in HCs and 941 differentially expressed proteins in MS patients. Of these proteins, 637 were differentially expressed in both HC and MS samples, whereas 353 and 304 proteins were exclusively differentially expressed upon CD4^+^ T cell activation in HCs and in MS patients, respectively (Fig. 1E). Of the 637 proteins differentially expressed in both groups, all proteins, except for pyruvate dehydrogenase and Late Endosomal/Lysosomal Adaptor, MAPK and MTOR activator 5, encoded by the *PDH6* and *LAMTOR5* genes, showed a change in expression in the same direction across the groups.

**Fig. 4:**
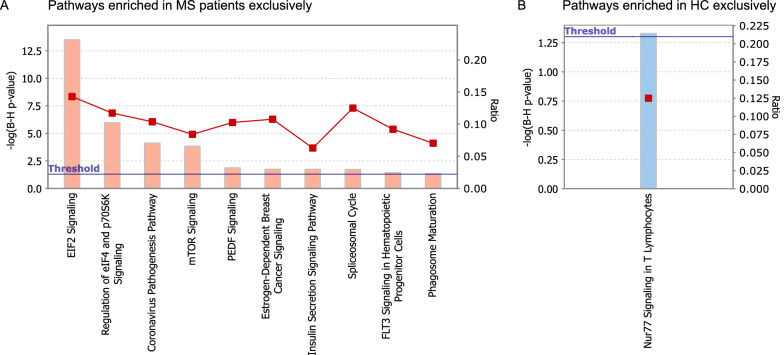
Biological pathways enriched upon CD4^+^ T cell activation. The graphs display the biological pathways enriched among proteins that are differentially expressed between unstimulated and stimulated CD4^+^ T cells exclusively in (**A**) MS patients (pink) or (**B**) HCs (light blue) after Benjamini-Hochberg (**B**–**H**) multiple testing correction (p-values seen on left y axis, blue line is marking the threshold level for significance). The red line with squares represents the ratio of the number of proteins in the data set of differentially expressed proteins divided by the number of proteins in the reference data set for that specific pathway (right y axis)

The IPA software was used for network analyses of proteins whose expression was affected by T cell activation exclusively in samples from MS patients or HCs. We identified enrichment in ten biological processes (Fig. 4A; − log(B-H p-value) > 1.3) among the proteins exclusively changed upon stimulation of CD4^+^ T cells from MS patients, whereas among the proteins exclusively changed upon activation of CD4^+^ T cells from HCs, we identified one biological process (Fig. 4B; − log(B-H p-value) > 1.3). The top four pathways (eIF2 signaling, regulation of eIF4 and p70S6K signaling, Coronavirus pathogenesis pathway, and mTOR signaling) identified among the proteins exclusively changed in MS patients corresponded to the top four pathways identified among the proteins whose expression were changed upon T cell activation in both groups (Table 6). Of note, the Nur77 signaling pathway identified among the proteins exclusively changed upon activation of CD4^+^ T cells from HCs has been shown to be a key regulator of T cell function by restricting activation, cell cycle progression, and proliferation [31].

**Table 6:** Pathways identified among proteins differentially expressed upon T cell activation in both MS and healthy control samples

Ingenuity canonical pathways	− log(p-value)
EIF2 signaling	33.90
Regulation of eIF4 and p70S6K signaling	15.00
Coronavirus pathogenesis pathway	9.48
mTOR signaling	8.18
Cytotoxic T Lymphocyte-mediated apoptosis of target cells	6.81
Antiproliferative role of TOB in T cell signaling	5.95
Superpathway of cholesterol biosynthesis	5.89
Protein ubiquitination pathway	5.68
OX40 signaling pathway	5.64
iCOS-iCOSL signaling in T helper cells	4.60
tRNA charging	4.58
Superpathway of serine and glycine biosynthesis I	4.36
Th1 pathway	4.17
Cholesterol biosynthesis I	3.90
Cholesterol biosynthesis II (via 24,25-dihydrolanosterol)	3.90
Cholesterol biosynthesis III (via Desmosterol)	3.90
CTLA4 signaling in cytotoxic T lymphocytes	3.87
PD-1, PD-L1 cancer immunotherapy pathway	3.74
Serine biosynthesis	3.54
Proline biosynthesis I	3.54
Type I diabetes mellitus signaling	3.52
Th1 and Th2 activation pathway	3.42
T helper cell differentiation	3.34
Glucocorticoid receptor signaling	3.31
Purine nucleotides De Novo biosynthesis II	3.10
Th2 pathway	3.08
Primary immunodeficiency signaling	2.97
Th17 activation pathway	2.97
BAG2 signaling pathway	2.97
Crosstalk between dendritic cells and natural killer cells	2.82
Epoxysqualene biosynthesis	2.75
Role of PKR in interferon induction and antiviral response	2.72
Antigen presentation pathway	2.65
Autoimmune thyroid disease signaling	2.58
Calcium-induced T lymphocyte apoptosis	2.51
Superpathway of geranylgeranyldiphosphate biosynthesis I (via Mevalonate)	2.32
Folate transformations I	2.29
Diphthamide biosynthesis	2.29
Cell Cycle: G1/S checkpoint regulation	2.29
Pyrimidine ribonucleotides De Novo biosynthesis	2.27
Methionine degradation I (to Homocysteine)	2.23
CD28 signaling in T helper cells	2.07
Cysteine biosynthesis III (mammalia)	2.06
Regulation of IL-2 expression in activated and anergic T lymphocytes	2.00
Proline biosynthesis II (from Arginine)	2.00
Trans, trans-farnesyl diphosphate biosynthesis	2.00
Allograft rejection signaling	1.97
FAT10 cancer signaling pathway	1.97
T cell exhaustion signaling pathway	1.95
IL-9 signaling	1.94
Role of JAK1, JAK2 and TYK2 in interferon signaling	1.91
Induction of apoptosis by HIV1	1.87
Assembly of RNA polymerase II complex	1.83
Hematopoiesis from pluripotent stem cells	1.83
Altered T cell and B cell signaling in rheumatoid arthritis	1.82
Mevalonate pathway I	1.81
Tetrahydrofolate salvage from 5,10-methenyltetrahydrofolate	1.79
Folate polyglutamylation	1.79
Nur77 signaling in T lymphocytes	1.76
Pyrimidine ribonucleotides interconversion	1.74
Systemic lupus erythematosus in T cell signaling pathway	1.73
Role of NFAT in regulation of the immune response	1.70
Graft-versus-host disease signaling	1.69
Activation of IRF by cytosolic pattern recognition receptors	1.64
CD27 signaling in lymphocytes	1.64
Lymphotoxin Œ ≤ receptor signaling	1.64
Cell cycle control of chromosomal replication	1.64
Arginine degradation VI (arginase 2 pathway)	1.63
Histidine degradation III	1.63
Citrulline biosynthesis	1.63
Zymosterol biosynthesis	1.63
Dendritic cell maturation	1.61
T cell receptor signaling	1.58
RAN signaling	1.56
Cdc42 signaling	1.55
PKCŒ∏ signaling in T lymphocytes	1.55
Aldosterone signaling in epithelial cells	1.53
FAT10 signaling pathway	1.49
Aryl hydrocarbon receptor signaling	1.46
Methylthiopropionate biosynthesis	1.38
Proline degradation	1.38
Acetyl-CoA biosynthesis III (from Citrate)	1.38
Asparagine biosynthesis I	1.38
Alanine biosynthesis III	1.38
Superpathway of methionine degradation	1.36
Caveolar-mediated endocytosis signaling	1.34

## Discussion

Genome-wide association studies have revealed 230 risk loci for MS, mostly located within or close to genes expressed in immune cells [7]. However, it remains to be analyzed whether genetic differences are translated into cell-specific expression profiles in samples from MS patients and HCs. Previous transcriptomic analyses of CD14^+^ monocytes, CD4^+^ and CD8^+^ T cells, indicated that CD4^+^ T cells were the most dysregulated cell type in MS among these three immune cells [32]. Transcriptomic profiling is frequently performed to identify genes and pathways of relevance for complex diseases such as MS. Given the lack of complete correlation between mRNA and protein copy numbers [24, 25], proteomic profiling enables an alternative or complementary approach for identification of disease relevant pathways. To our knowledge, we were the first to perform proteomic profiling of purified immune-cell subsets from MS patients. Using electrospray liquid chromatography-tandem mass spectrometry, we were able to identify aberrant protein expression in freshly purified T cells, i.e. CD4^+^ and CD8^+^ T cells, from MS patients as compared to HCs [26]. In the current study, we used the same technique for proteomic profiling of CD4^+^ T cell samples left unstimulated or stimulated for 24 h in vitro through the TCR, to analyze protein dysregulation during T cell activation in MS. Our PCA analyses showed separated clusters of MS patients and HCs in both the unstimulated and stimulated samples. Moreover, two distinct clusters appeared among the stimulated CD4^+^ T cell samples within the MS group: the samples from three MS patients were clearly separated from the other 17 MS patients. However, these three MS patients were not clinically different from the rest of the group. Even though cell purity, cell viability and activation status were comparable in all samples, we cannot exclude that other cellular phenotypes, e.g. different CD4^+^ T cell subpopulation frequencies, could be causing the separation of the three samples from the remaining 17 in the PCA plot.

We identified novel proteins that were differentially expressed in response to activation in samples from MS patients as compared to HCs. Furthermore, we found that the proteins, whose expression was changed upon T cell activation, were enriched for proteins encoded by MS susceptibility genes. These findings confirmed the importance of CD4^+^ T cell activation for MS pathogenesis. As the included patients already had developed MS, it remains to be shown whether this aberrant response contributes to developing MS or rather is a consequence of the ongoing disease. Of note, all included MS patients were untreated and clinically stable at the time of sample collection, excluding the possibility for disease modifying treatment having affected the T cells used in this study.

There is little overlap between the findings from this study and Berge et al., 2019 [26], but both studies were relatively low powered due to the small sample sizes. To rule out findings attributable to low sample size, a validation analysis was performed in the current study and confirmed the protein dysregulation observed in MS patients. Furthermore, even though eight samples (four MS and four HCs) were obtained from the same donors as included in [26], the experimental set ups were different between the two studies. In our previous study [26], the CD4^+^ T cells were prepared for mass spectrometry directly after cell purification to investigate their status in MS patients. On the contrary, for this study, live cells were stored on liquid nitrogen prior to thawing and cell cultivation for 24 h in the presence or absence of stimulating antibodies to investigate T cell behavior upon activation. All samples included in this study were treated equally, and there was no difference between the two groups in cell viability (Fig. 2B) or cell activation, as measured by cell surface expression of CD69 using flow cytometry (Fig. 2A). Using our stimulation protocol, cells were triggered through the TCR and CD28 co-receptor. Therefore, all T cells in the culture, independent of specificity and binding strength, were likely to be activated, ruling out the possibility of a different TCR repertoire in the MS population. Through proteomic profiling of stimulated cells, we identified MS-associated proteins that were hitherto not identified with the current available approaches, i.e. global DNA methylation analyses or RNA sequencing, performed in untreated immune cell subsets, full blood or in PBMCs [10–23].

There were only two proteins differentially expressed between MS patients and HCs in both the unstimulated and stimulated samples, i.e. diphthine:ammonia ligase (also called diphthamide synthetase) encoded by *DPH6* and enhancer of polycomb homolog 1 encoded by *EPC1*. Diphthamide synthetase catalyzes the conversion of histidine to diphthamide for regulation of the translation factor EEF2 [33], which controls neurological processes [34], but with hitherto no known role in autoimmunity. Enhancer of polycomb homolog 1 is a transcriptional regulator [35] with no known function in T cells and was also one of two proteins differentially expressed in all the 100 iterations performed with the stimulated samples. Another protein differentially expressed in all the 100 iterations and the top hit of the main analysis in the stimulated samples (log2 fold change = 5.35), was Grb2-related adapter protein encoded by *GRAP*. Of note, Grb2-related adapter protein 2 encoded by the MS susceptibility gene *GRAP2* was expressed at higher levels in CD4^+^ T cells from MS patients as compared to HCs in our previously published proteomic analyses [26]. The Grb2 family of adapter proteins has been shown to interact with the activated T cell costimulatory receptor CD28 [36] and to be involved in Erk-MAP kinase activation in human B cells [37]. Moreover, the *GRAP* gene is primarily expressed in human thymus and spleen [38], and it negatively regulates TCR-elicited proliferation and interleukin-2 induction in murine lymphocytes [39]. Identification of these adapters in our proteomic approaches suggests further investigation of the Grb2 family of adaptor proteins in MS.

Among the differentially expressed proteins between MS patients and HCs, three proteins have previously been suggested to play a role in MS pathogenesis: (1) tyrosine kinase 2 (TYK2), (2) protein tyrosine phosphatase non-receptor type 2 (PTPN*2*), and (3) DNA polymerase subunit gamma-1 (POLG*)*. In our data set, TYK2 was slightly upregulated in unstimulated samples from MS patients (log2 fold change = 1.14). An exonic *TYK2* variant (rs34536443) has been shown to associate with increased MS risk [7], and the presence of the protective allele at rs3453443 resulted in reduced TYK2 kinase activity in T cells and a shift in the cytokine secretion profile favoring Th2 development, but did not modify TYK2 expression when measured by Western blotting [40]. With a minor allele frequency of 0.01423 (www.snpedia.com) for the MS associated rs34536443 SNP in *TYK2* and the limited sample size in the presented study, it is unlikely that the genotype of this SNP underlies the difference in TYK2 expression between the two groups. PTPN2 has previously been linked to MS as a microRNA, i.e. miR-448, that was upregulated in PBMC and cerebrospinal fluid (CSF) from MS patients, promoted IL-17 production directly through PTPN2, thereby contributing to development of an autoinflammatory immune environment. However, being a direct target of miR-448, *PTPN*2 expression was reduced in PBMC and CSF from MS patients [41], whereas we observed a small increase in stimulated CD4^+^ T cells from MS patients (log2 fold change = 0.85). Nevertheless, the experimental set-up and the biological materials were different in the two studies. In our analyses, we were able to detect cell-specific differences, which could be convoluted when analyzing heterogeneous samples such as PBMCs or CSF. POLG expression was reduced in stimulated CD4^+^ T cells from MS patients (log2 fold change = − 1.35) as compared to HC samples. Genetic variants in the *POLG* gene have been associated with familiar MS [42]. In a smaller genetic study, *POLG* was suggested as an MS susceptibility gene [43], but it did not reach genome-wide significance in the latest MS GWAS [7].

As MS is an autoimmune disease, it is not a surprise that proteins expressed from MS susceptibility genes are enriched among the proteins that change expression upon T cell activation, highlighting the importance of this process in MS. Findings from our previous proteomic study [26] also pointed to the importance of T cell activation, as the differentially expressed proteins between CD4^+^ T cells from MS patients and HCs were enriched in pathways related to T cell activation. In the current study, most proteins that were induced or inhibited upon CD4^+^ T cell stimulation were overlapping in samples from MS patients and HCs. However, there were sets of proteins that were selectively regulated in one group only. Pathway analyses showed that proteins with changes in expression upon T cell activation in the MS group only correspond to pathways also identified among the proteins changed upon T cell activation in both groups, including pathways of translation initiation and immune response (eIF2 and eIF4) and cell survival and proliferation (mTOR). Interestingly, pathway analysis showed that proteins with changes in expression upon T cell activation in the HC group only were enriched for the Nur77 pathway. This signaling pathway limits aberrant effector T cell responses and impedes the development of T cell-mediated inflammatory diseases such as autoimmune disorders [31]. Nur77-dependent regulation of inflammation occurs by inhibiting the nuclear factor-κB (NF-κB) pathway [44]. Deficiencies in the Nur77 pathway increase NF-κB activity and, consequently inflammation in murine models [45]. Furthermore, the role of NF-κB activation in MS pathogenesis has been confirmed in several studies and drugs targeting this pathway already gained FDA approval for MS treatment [46]. In line with these findings, our data suggest that in contrast to in HCs, the Nur77 pathway is unchanged upon T cell activation in MS patients possibly leading to increased NF-κB activation and inflammation. The molecular link between Nur77 dysregulation and MS needs further verification in a bigger and independent cohort prior to thorough functional analyses to elucidate the role of the Nur77 pathway in the development of MS and to evaluate whether this pathway could be used as a diagnostic and/or therapeutic target.

In the current study, we examined one immune cell subtype from blood, CD4^+^ T cells, which provided a detailed insight into one specific immune cell subtype with a likely role in MS. However, it should be noted that CD4^+^ T cells can be further divided into subclasses and consequently differences in subtypes of CD4^+^ T cells, such as Th17 or regulatory T cells, might not be detected, as these signals may be concealed by signals from the more abundant CD4^+^ T cells subtypes. Although we have identified novel proteins of potential importance for MS, further studies are needed to validate and verify the biological impact of selected proteins and pathways in T cells.

## Conclusions

In summary, using electrospray liquid chromatography-tandem mass spectrometry for analyses of in vitro stimulated CD4^+^ T cells from MS patients and HCs, we were able to identify aberrant regulation of novel proteins, hitherto not identified through other approaches. Proteins encoded by MS susceptibility genes are enriched among proteins that change in abundance upon T cell activation, and through pathway analyses, we have identified enrichment of induced proteins from the Nur77 pathway in HC samples only.

## Data Availability

The datasets generated and analyzed during the current study are available via ProteomeXchange with identifier PXD028702.

## Abbreviations

MS: Multiple sclerosis
HC: Healthy control
CNS: Central nervous system
GWAS: Genome wide association study
SNP: Single nucleotide polymorphism
PBMC: Peripheral blood mononuclear cell
RRMS: Relapsing–remitting multiple sclerosis
TCR: T cell receptor
EDSS: Extended disability status scale
FITC: Fluorescein isothiocyanate-conjugated
DMSO: Dimethyl sulfoxide
DTT: Dithiothreitol
LC–MS/MS: Electrospray liquid chromatography-tandem mass spectrometry
HCD: Higher-energy collision dissociation
NCE: Normalized collision energy
Mass spec: Mass spectrometry
PCA: Principal component analysis
B-H: Benjamini-Hochberg
IPA: Ingenuity pathway analysis
TYK2: Tyrosine kinase 2
PTPN2: Protein tyrosine phosphatase non-receptor 2
POLG: DNA polymerase subunit gamma-1
CSF: Cerebral spinal fluid
NF-κB: Nuclear factor-κB
